# Supplementary material for: Application of artificial neural networks and genetic algorithm to predict and optimize greenhouse banana fruit yield through nitrogen, potassium and magnesium
Source: PLoS One. 2022 Feb 14;17(2):e0264040. doi: 10.1371/journal.pone.0264040 (PMC8843134; doi:10.1371/journal.pone.0264040)
Supplement: Attachment — Submitted filename: Response to Reviewers.docx [file pone.0264040.s001.docx]

**Dear Dr. Ravindran,**

We would like to thank you for the letter, and the opportunity to submit a revised copy of this manuscript. We would also like to take this opportunity to express our thanks to the reviewers for the comments. We believe have resulted in an improved revised manuscript, which you will find uploaded alongside this document. The manuscript has been revised to address the reviewer comments, detailed in your letter properly as explained below.

**Reviewer #1:**

1. There is no need to write the complete form of the words after being introduced as abbreviated forms. Please mention the complete form on in the first appearance of the words in the main text.

- The words were written in their complete form for the first time where they were mentioned and in the rest parts we used only them as abbreviate form.

2. You should mention the unit of measurement (gr etc.) for first three columns of Table 1.

- The unit of the two first characters were written in the table 1, the third characters does not unit, its number.

3. Choose a better title for Table 2. Please pay close attention to the English language proficiency.

- The title of table was rewrite.

4. Kindly add an appropriate legend for column “prediction” in Table 3. What is the min, max and optimal number? Is there a specific range? Please provide these kind of information.

- To clarify we changed the title of Table 3, the values are optimal amount of the three fertilizers used in this study for the measured characters.

5. Please provide more in-depth caption for Fig.2 and Fig.3. Describe the correlations in Fig.2 and clustering in Fig.3 in more details.

- The captions of the figures were written in more in-depth.

**Reviewer #2:**

A. There are some repeated words and sentences please check them and rewrite.

- The sentences were rewritten

B. please check the first paragraph of conclusion there are also repasts.

- The first paragraph in conclusion section was revised.

Best regards,

Corresponding author,
